# Author Correction: Buried deep freshwater reserves beneath salinity-stressed coastal Bangladesh

**DOI:** 10.1038/s41467-026-69228-z

**Published:** 2026-02-04

**Authors:** Huy Le, Kerry Key, Michael S. Steckler, Nafis Sazeed, Mark Person, Anwar Bhuiyan, Mahfuzur R. Khan, Kazi M. Ahmed

**Affiliations:** 1 Department of Earth and Environmental Sciences, Columbia University, New York, NY USA; 2 Lamont-Doherty Earth Observatory, Columbia University, New York, NY USA; 3 Now at Deep Blue Geophysics, LLC, Los Angeles, CA USA; 4 New Mexico Institute of Mining and Technology, Socorro, NM USA; 5 Department of Geology, University of Dhaka, Dhaka, Bangladesh

**Keywords:** Geophysics, Hydrogeology

Correction to: *Nature Communications* 10.1038/s41467-025-65770-4, published online 28 November 2025

In the version of this article initially published, the images shown as Fig. 2b, c were earlier, incorrect versions, where panel b originally showed the salinity model without incorporating seismic data to derive porosity estimation, while in panel c, R1 and R2 resistor labels were incorrect. The figure has been updated in the HTML and PDF versions of the article. For comparison, the original figure is available as Supplementary information accompanying this amendment.

## Supplementary information

Original Fig. 2

