# Supplementary material for: Buried deep freshwater reserves beneath salinity-stressed coastal Bangladesh
Source: Nat Commun. 2025 Nov 28;16:10740. doi: 10.1038/s41467-025-65770-4 (PMC12663281; doi:10.1038/s41467-025-65770-4)
Supplement: Supplementary file 1 — Supplementary Information [file 41467_2025_65770_MOESM1_ESM.pdf]

Supplementary Information

Buried deep freshwater reserves beneath salinity-stressed coastal Bangladesh

Huy Le[1*], Kerry Key[2,3*], Michael S. Steckler[2*], Nafis Sazeed[4], Mark Person[4], Anwar Bhuiyan[5], Mahfuzur R. Khan[5], and Kazi M. Ahmed[5]

[1]Department of Earth and Environmental Sciences, Columbia University, Palisades, New York, USA

[2]Lamont-Doherty Earth Observatory, Columbia University, Palisades, New York, USA

[3]Now at Deep Blu Geophysics, LLC, Los Angeles, CA 90048, USA

[4]New Mexico Institute of Mining and Technology, Socorro, New Mexico, USA

[5]Department of Geology, University of Dhaka, Dhaka, Bangladesh

*****Corresponding author email: hdl2115@columbia.edu, kkey@deepbluegeophysics.com, steckler@ldeo.columbia.edu

Supplementary Figures

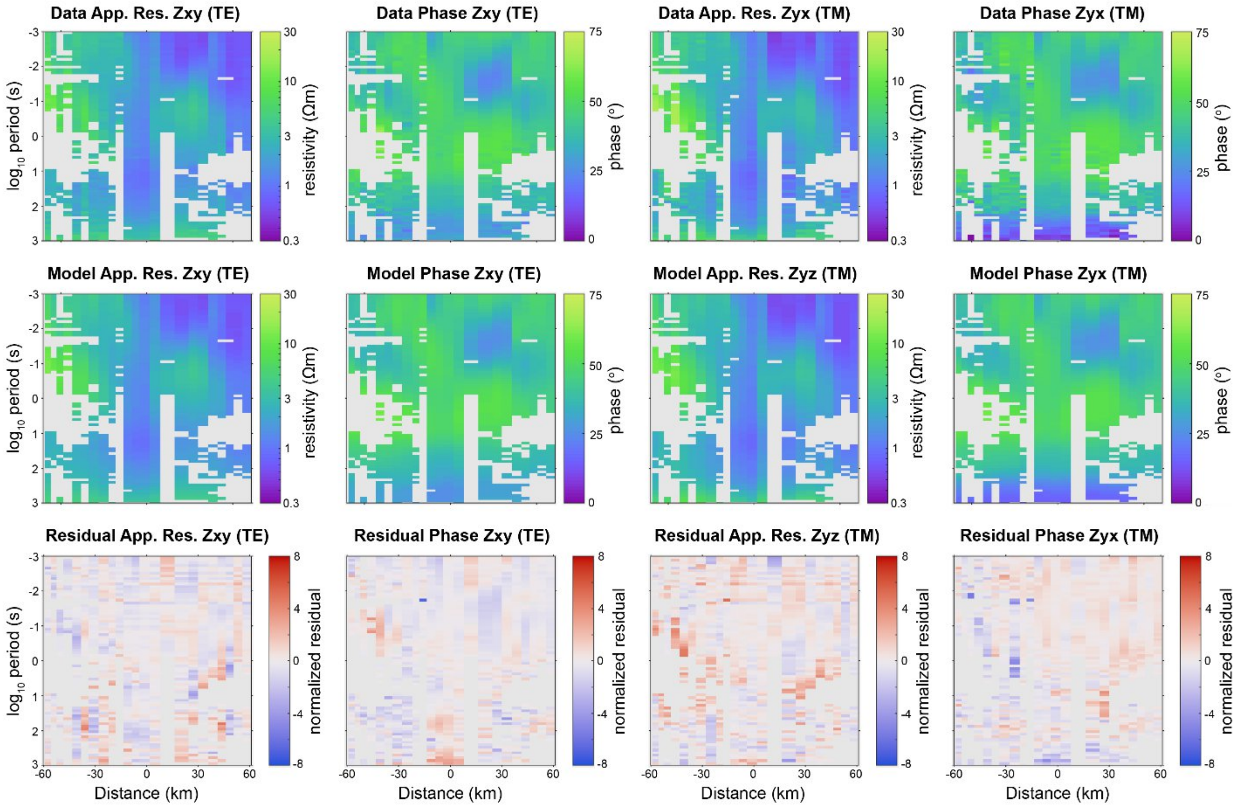

Supplementary Figure 1. MT data and model responses. Pseudosections showing MT data (top), inversion model responses (middle), and normalized residuals (bottom) as apparent resistivity and phase for the transverse electric (TE) and transverse magnetic (TM) modes.

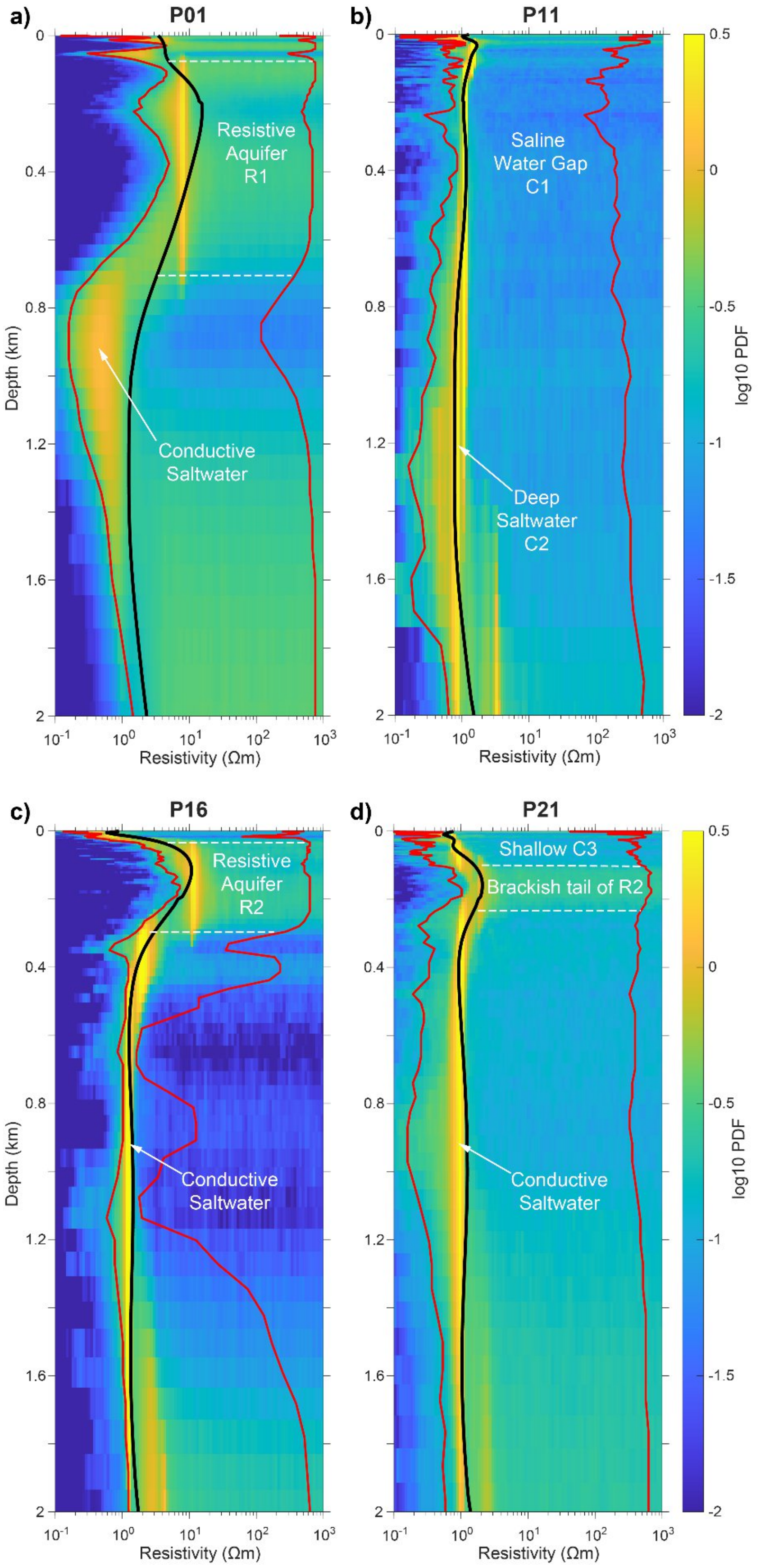
a)
P01
Resistive Aquifer R1
Conductive Saltwater
Depth (km)
Resistivity (Ωm)
b)
P11
Saline Water Gap C1
Deep Saltwater C2
Resistivity (Ωm)
log10 PDF
c)
P16
Resistive Aquifer R2
Conductive Saltwater
Depth (km)
Resistivity (Ωm)
d)
P21
Shallow C3
Brackish tail of R2
Conductive Saltwater
Resistivity (Ωm)
log10 PDF

Supplementary Figure 2. Bayesian inversion results. Bayesian 1D inversion models of MT data from stations P01 (**a**), P11(**b**), P16 (**c**), and P21 (**d**) (see Fig. 2). Shaded colors show the posterior probability density functions (PDF) obtained from the MT responses. Warm and cold colors indicate the high and low probability of resistivity, respectively. The region between the left (5th percentile) and the right (95th percentile) red curves indicates the 90 percent credible interval where resistivity values are more likely to match MT data[1]. The black curve exhibits an extracted 1D resistivity profile from the 2D regularized inversion model from Fig. 2a. The high posterior PDF of resistors R1 and R2 at P01 (panel **a**) and P16 (panel **c**) agree with the 2D regularized model, whereas the Bayesian 1D model at P11 (**b**) confirms the saline water gap C2. Note that the 95th percentiles are close to the prior upper bound (10^3 Ωm). This reflects that MT data are unable to resolve the magnitude of highly resistive features due to response saturation.

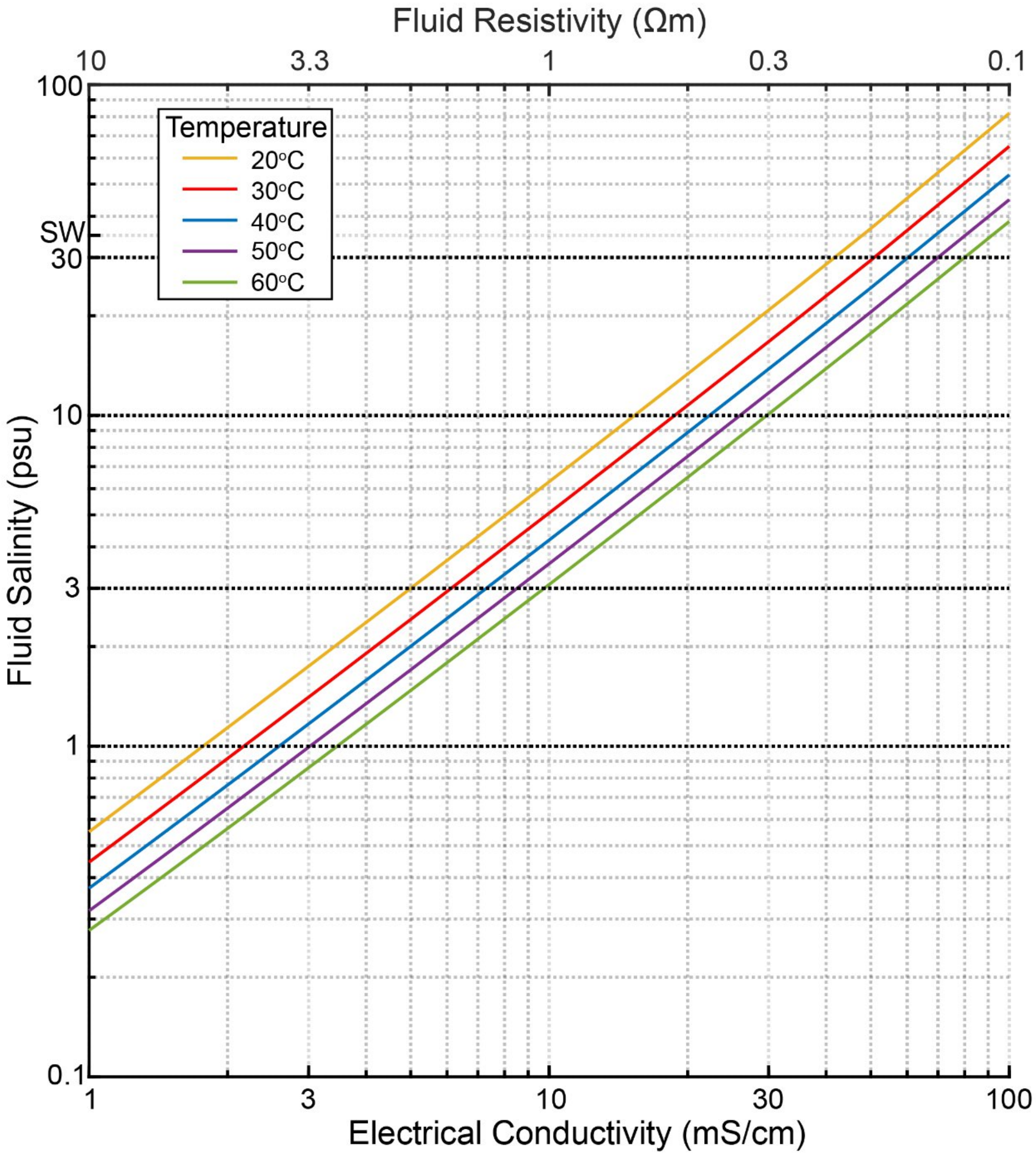

Supplementary Figure 3. Relationship between fluid salinity in PSS78 scale[2] and conductivity at varied temperatures. Assuming that the pressure effect is negligible, salinity in PSS78 scale only depends on fluid electrical conductivity and temperature. Pressure equals to 0 dbars in the conversion. Note that higher temperature is associated with lower salinity at specific fluid conductivity.

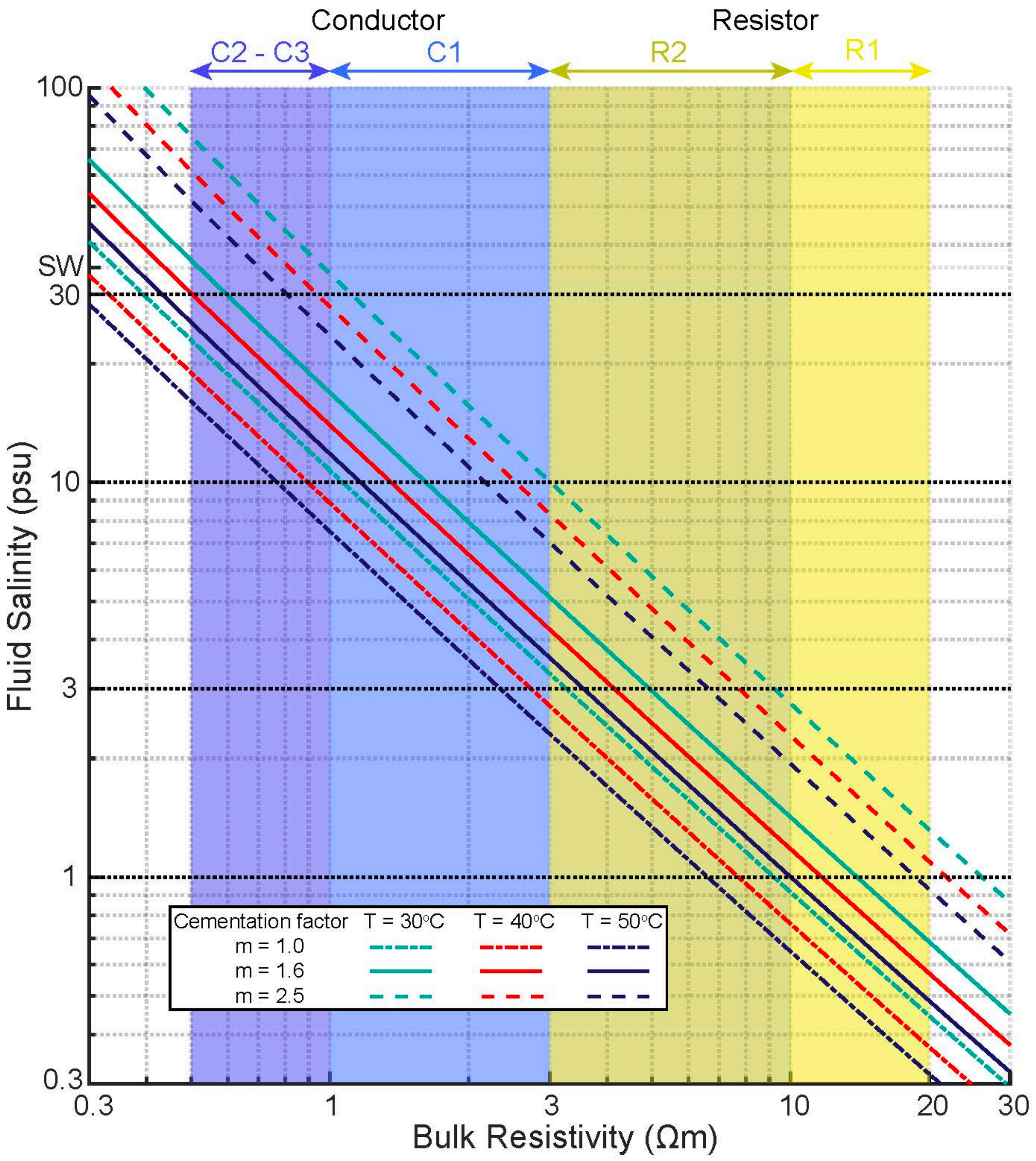

Supplementary Figure 4. Pore fluid salinity varies with bulk resistivity, temperature, and cementation factor. Salinity is calculated as a function of bulk resistivity (ρ_b), temperature (T) and cementation factor (m) by using Archie's law[3] and PSS78 scale[2] with fixed pressure and porosity: P = 0 dbars and ϕ = 50%. High bulk resistivity, high temperature, and low cementation factor correspond with fresh and low-salinity fluids, and vice versa.

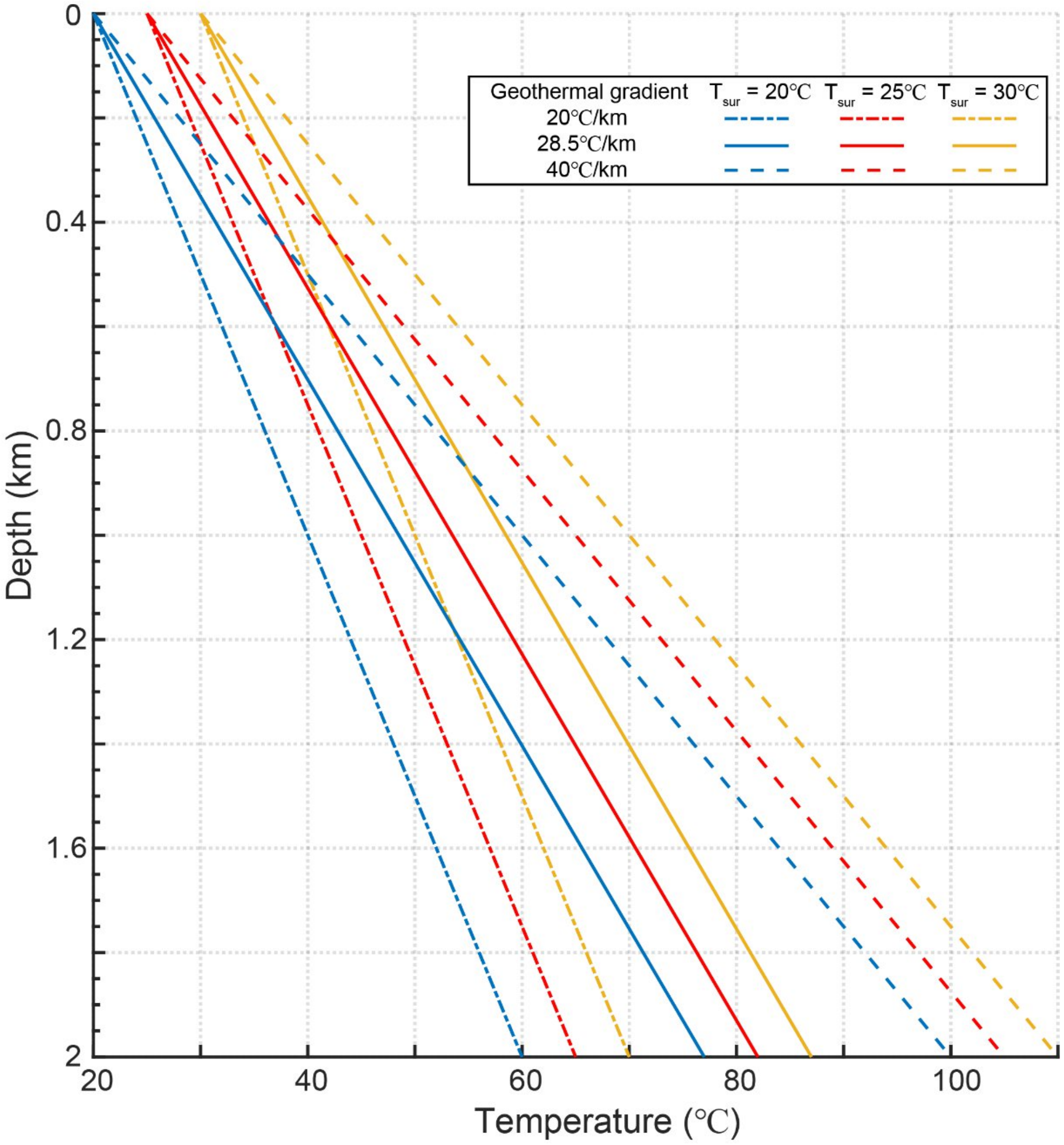

Supplementary Figure 5. Fluid temperature profile as a function of depth with varied surface temperature and geothermal gradients. Our preferred model uses the T_s = 25°C representing the surface temperature of water in the tropical regions and a geothermal gradient of 28.5°C/km for the SW Bangladesh[4]. We assume that the fluid temperature of groundwater is in equilibrium with the surrounding sediments.

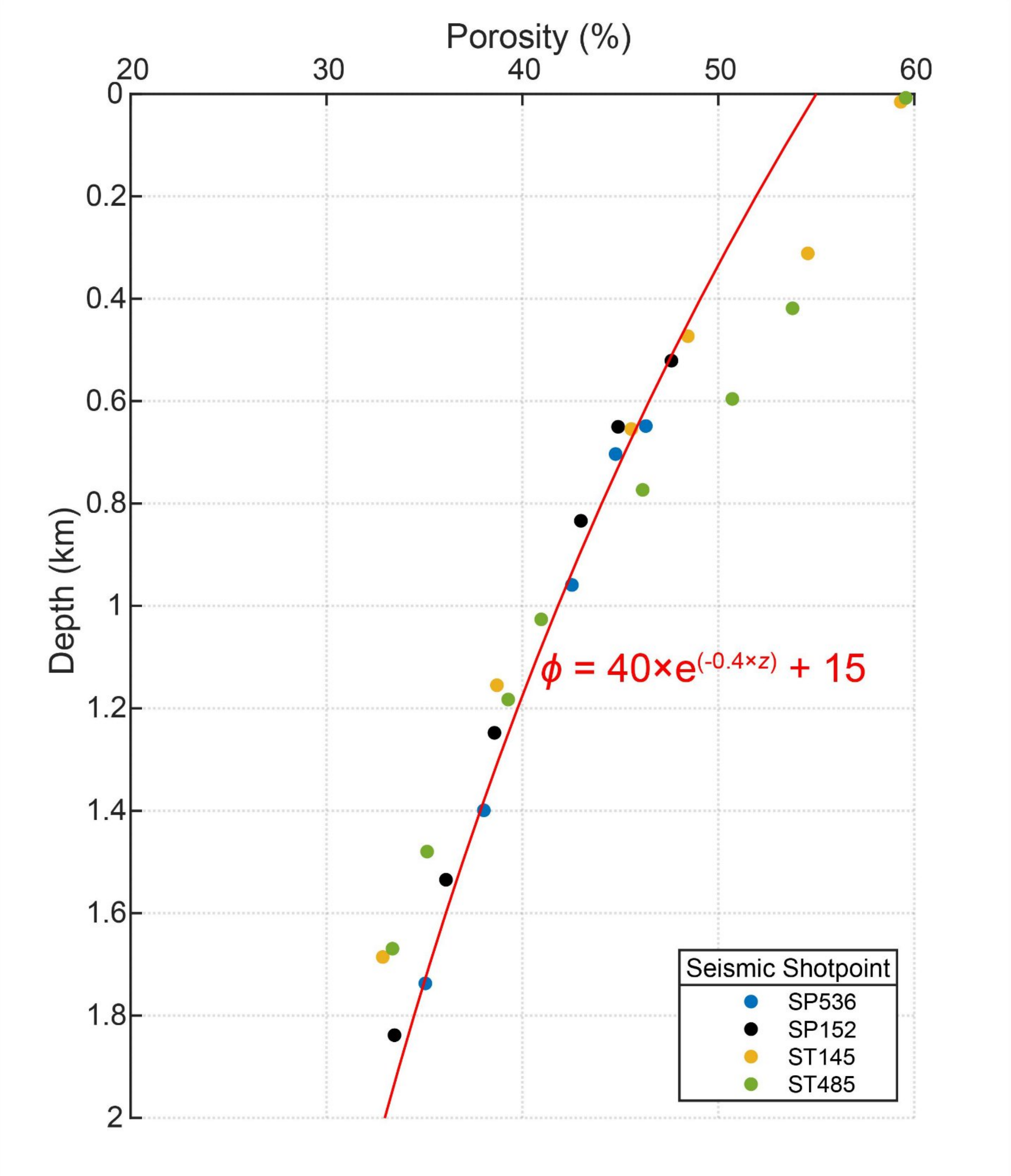

Supplementary Figure 6. Estimated porosity versus depth from multichannel seismic reflection surveys. Diamonds show porosity derived from line PK-15; circles show porosity derived from line LRU-08 (see Supplementary Table 1). The curve illustrates the fitting line using empirical Athy's law[5] and shows the porosity model used for estimating salinity model in Fig. 2b. The porosity equation is ϕ (%) = $40 \times e^{(-0.4 \times z)} + 15$. Our preferred model uses an initial surface porosity $\phi_o + \phi_{min}$ of 55%, the minimum porosity ϕ_{min} of 15%, and a compaction factor of 0.4 km^{-1}.

Supplementary Table 1. Derived porosity from seismic data (see Fig. 1b for location).

The recorded time for all shotpoints from Line PK-15 and LRU-08 is two-way travel time from multi-channel seismic reflection. We applied a depth conversion equation[6] from travel time and seismic velocity:

$$Z = (e^{C \times T/2} - 1) \times (V_o/C) \qquad (4)$$

Where Z is the depth (km) and T is the two-way travel time (s). V_o and C are the y-intercept and slope of fitting linear equation for seismic velocity (km/s) and travel time (s).

For estimating density, we used the polynomial regression fit of Nafe-Drake curve relating P-wave velocity to density[7]:

$$\rho = 1.6612 \times V_p - 0.4721 \times V_p^2 + 0.0671 \times V_p^3 - 0.0043 \times V_p^4 + 0.000106 \times V_p^5 \qquad (5)$$

Then we calculated porosity using the constraint:

$$\phi = (2.68 - \rho) / (2.68 - 1.03) \times 100$$

where ϕ is the porosity (%), and ρ is the density (g/cm^3). The porosity constraint here assumes that density is a linear mixture of seawater (ρ = 1.03 g/m^3) and rock with a grain density of 2.68 g/cm^3.

Shotpoint	Travel time (s)	Velocity (km/s)	Depth (km)	Density (g/cm^3)	Porosity (%)
SP536 Line PK-15	0.747	2.024	0.649	1.916	46.30
	0.803	2.038	0.703	1.942	44.76
	1.053	2.173	0.959	1.978	42.53
	1.444	2.375	1.399	2.053	38.03
	1.716	2.528	1.737	2.102	35.04
	2.002	2.733	2.124	2.160	31.53
	2.295	2.917	2.555	2.205	28.78
	2.542	3.055	2.949	2.236	26.93
	2.961	3.298	3.688	2.283	24.04
	3.173	3.465	4.099	2.312	22.27
	3.679	3.784	5.191	2.362	19.25
	0.564	1.976	0.521	1.895	47.60

SP152 Line PK-15	0.692	2.078	0.650	1.939	44.88
	0.867	2.154	0.834	1.971	42.98
	1.235	2.349	1.248	2.044	38.57
	1.471	2.472	1.535	2.084	36.10
	1.706	2.616	1.838	2.128	33.47
	2.072	2.827	2.349	2.184	30.08
	2.394	2.974	2.840	2.218	27.99
	2.853	3.172	3.614	2.259	25.48
	3.135	3.288	4.137	2.282	24.15
ST145 Line LRU-08	0.02	1.607	0.015	1.701	59.32
	0.38	1.745	0.311	1.780	54.57
	0.56	1.946	0.473	1.881	48.44
	0.75	2.052	0.654	1.928	45.56
	1.22	2.343	1.155	2.041	38.70
	1.65	2.651	1.686	2.138	32.87
	2.03	2.915	2.221	2.205	28.80
	2.24	3.074	2.547	2.240	26.69
	2.40	3.186	2.811	2.262	25.32
	2.59	3.399	3.143	2.301	22.95
	3.17	3.760	4.295	2.359	19.47
	3.37	3.894	4.746	2.378	18.28
	3.77	4.106	5.742	2.408	16.48
	4.51	4.478	7.968	2.459	13.38
	4.99	4.723	9.730	2.494	11.30
ST145 Line LRU-08	0.01	1.600	0.008	1.697	0.5957
	0.50	1.769	0.419	1.792	0.5379
	0.69	1.868	0.596	1.843	0.5072
	0.87	2.030	0.773	1.919	0.4614
	1.11	2.240	1.026	2.004	0.4096
	1.25	2.316	1.183	2.032	0.3927
	1.50	2.523	1.480	2.100	0.3513
	1.65	2.622	1.669	2.129	0.3337
	1.90	2.756	2.006	2.166	0.3116
	2.00	2.841	2.148	2.187	0.2987
	2.16	2.932	2.385	2.209	0.2857
	2.46	3.158	2.862	2.257	0.2565
	2.95	3.454	3.744	2.311	0.2239
	3.75	3.873	5.507	2.375	0.1846
	5.00	4.561	9.313	2.471	0.1269

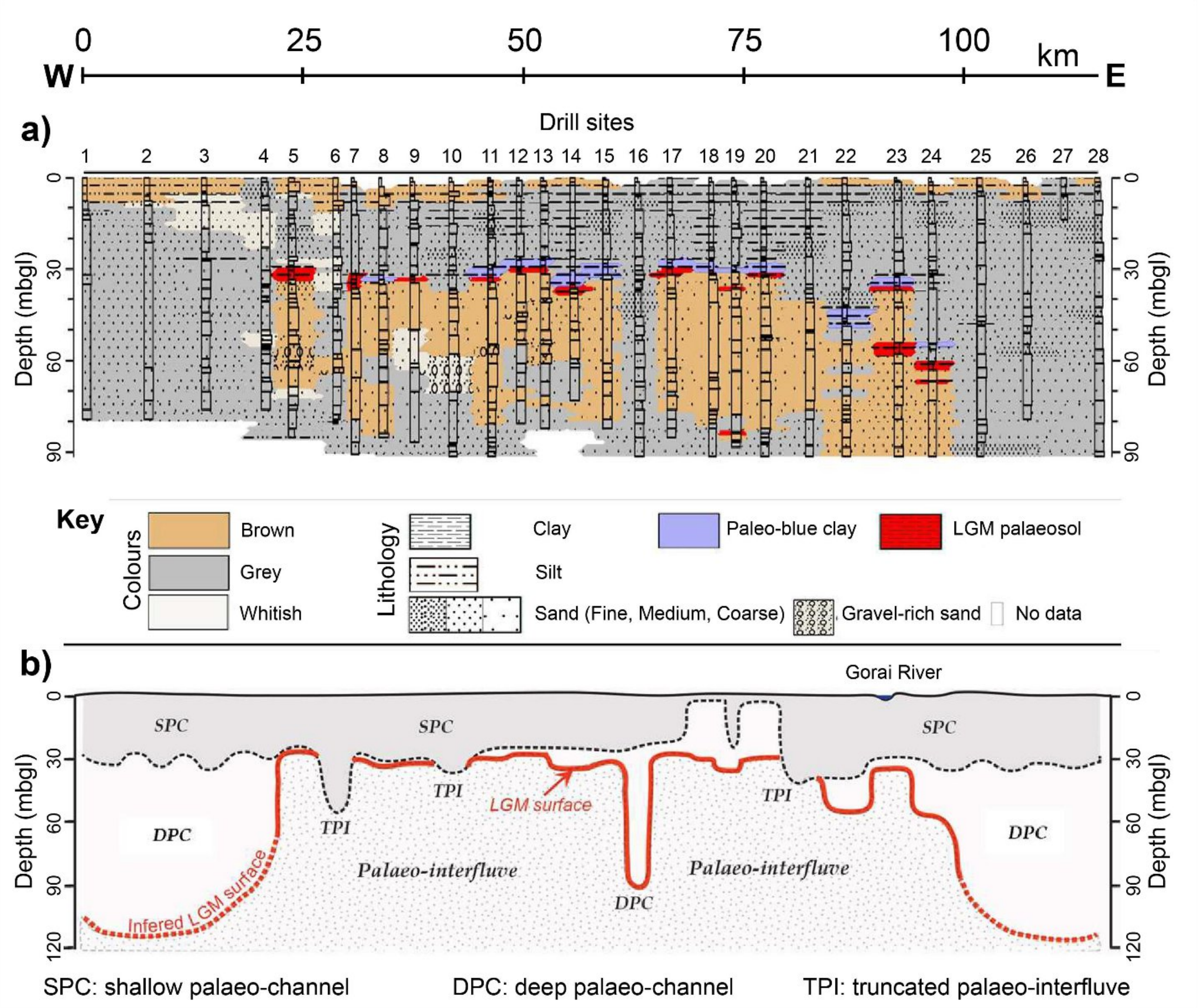

Supplementary Figure 7. A shore-parallel geological cross-section at 23.4ºN showing LGMP distribution from Hoque et al. (2014)[8]. (a) Inferred geological section based on drilling data. (b) Schematic interpretation of the section from the well data showing the LGMP in the palaeo-interfluve between two large incised DPCs associated with the Ganges and Brahmaputra Rivers. (Modified from Figure 5 in Hoque et al. (2014). "Sources of low-arsenic groundwater in the Bengal Basin: investigating the influence of the last glacial maximum palaeosol using a 115-km traverse across Bangladesh". Licensed under CC BY 4.0).

Overpressure Fluid-Porosity Test

Computed formation resistivity inversions are sensitive to porosity variations. The porosity-depth decay patterns inferred from seismic survey data appear to be under-compacted and are likely influenced by excess pressure formation. To assess this, we used an effective stress form of Athy's Law[9] to calculate porosity decay with depth:

$$\phi = \phi_o exp\left[-\beta\sigma_e\right] \quad (6)$$

where ϕ is porosity, ϕ_o is the porosity at the sediment-water interface (0.6), β is sediment compressibility (3.3×10^{-8} Pa^{-1}), σ_e is effective stress ($\sigma_e = \sigma_v - P$; Pa); P is pore-fluid pressure (Pa), and σ_v is the vertical load ($\sigma_v = gd\left[\phi\rho_f + (1-\phi)\,\rho_s\right]$; Pa), g is the gravity constant, d is depth (m), ρ_s is the sediment density, and ρ_f is fluid density. The sediment load is 23 MPa/km. Hydrostatic pressure is 10 MPa/km.

The governing equation we used to represent overpressure generation during subsidence and sedimentation is given by[10]:

$$S_s\left[\frac{\partial h}{\partial t} - \frac{\rho_s - \rho_f}{\rho_f}\frac{\partial L}{\partial t}\right] = \frac{\partial}{\partial z}\left[\frac{k\rho_f g}{\mu_f}\frac{\partial h}{\partial z}\right] \quad (7)$$

where S_s is specific storage (10^{-4} m^{-1}), L is the sedimentary column length, $\frac{\partial L}{\partial t}$ is the sedimentation rate (1.2 mm/yr), h is hydraulic head (h=P/(ρ_fg) +z), P is pore pressure, z is elevation, k is permeability, ρ_f is fluid density, μ_f is fluid viscosity. Equation (7) assumes a loading efficiency of 1[11]. We used a long-term sedimentation rate of 1.2 mm/year for the Bengal delta, which is conservative[12], indicate sedimentation rates within the Bengal delta are between 2-4 mm/yr).

Typically, in a prograding delta, sediments in the upper 1-2 km are coarse-grained and are underlain by finer grained silt and clay deposits[9]. To represent permeability decay with depth

we related the $\log_{10}$ permeability to porosity[13] assuming two different permeability-porosity models:

$$\log_{10}(k) = 5\times\phi - 15 \qquad (8)$$

$$\log_{10}(k) = 5\times\phi - 20 \qquad (9)$$

Equation (8) is referred to herein as the high permeability scenario, while equation (9) is referred to as the low permeability scenario. Permeability decay with depth/effective stress for the two scenarios are presented in Supplementary Fig. 7c.

We ran the *MATLAB* based overpressure model for 5 million years using a constant sedimentation rate of 1.2 mm/yr. Computed pressures changes with depth for the low and high permeability scenarios are presented in Supplementary Fig. 7a. The low permeability scenario produced overpressures at 3 km depth of about 50% of lithostatic levels (blue solid line; 50 MPa above hydrostatic levels). The high permeability scenario generated nearly hydrostatic pressures (red dashed line). For the low permeability scenario, pore spaces were held open by relatively high fluids pressures (red dashed line in Supplementary Fig. 7b) and lower effective stress levels (σ_e). For the high permeability scenario porosity (blue line in Supplementary Fig. 7b) decay of porosity with depth was more pronounced. The low permeability scenario closely matches the observed data in Supplementary Fig. 7b consistent with the empirical model. Overpressures with depth within the Bengal Delta are well-known[4] and not surprising given the high sedimentation rates and silt dominated sediments at depth.

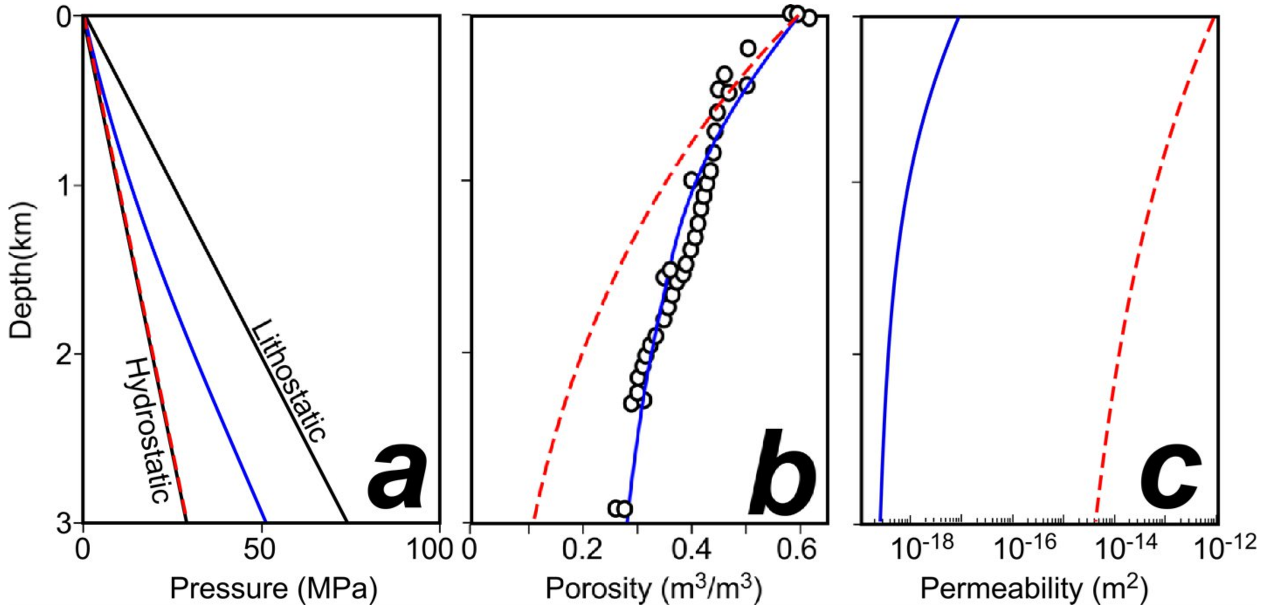

Supplementary Figure 8. Overpressure fluid-porosity test. (a) Comparison of fluid pressures for the low permeability (blue line) and high (red dashed line) permeability scenarios from the compaction model. Solid black lines denote lithostatic and hydrostatic pressures curves. (b) Computed porosity using Athy's law[5] for the low and high permeability model runs. White circles denote porosity estimated from the seismic data shown in Supplementary Fig. 6. (c) Permeability vs depth for the low and high permeability model runs.

Supplementary References

1. Blatter, D., Key, K., Ray, A., Gustafson, C., & Evans, R. Bayesian joint inversion of controlled source electromagnetic and magnetotelluric data to image freshwater aquifer offshore New Jersey. *Geophysical Journal International*, **218**(3), 1822-1837. (2019).

2. Lewis, E. L., & Perkin, R. G. Salinity: Its definition and calculation. *Journal of Geophysical Research: Oceans*, 83(C1), 466-478. (1978).

3. Archie, G. E. The electrical resistivity log as an aid in determining some reservoir characteristics. *Transactions of the AIME*, 146(01), 54-62. (1942).

4. Zahid, K. M., & Uddin, A. Influence of overpressure on formation velocity evaluation of Neogene strata from the eastern Bengal Basin, Bangladesh. *Journal of Asian Earth Sciences*, 25(3), 419-429. (2005).

5. Athy, L. F. Density, porosity, and compaction of sedimentary rocks. *AAPG Bulletin*, **14**(1), 1-24. (1930).

6. Sorlien, C. C. et al. Uniform basin growth over the last 500 ka, North Anatolian Fault, Marmara Sea, Turkey. *Tectonophysics,* **518**, 1-16. (2012).

7. Brocher, T. M. Empirical relations between elastic wavespeeds and density in the Earth's crust. *Bulletin of the seismological Society of America*, **95**(6), 2081-2092. (2005).

8. Hoque, M. A., McArthur, J. M., & Sikdar, P. K. Sources of low-arsenic groundwater in the Bengal Basin: investigating the influence of the last glacial maximum palaeosol using a 115-km traverse across Bangladesh. *Hydrogeology Journal*, **22**(7), 1535-1547. (2014).

9. Dugan, B., & Flemings, P. B. Overpressure and fluid flow in the New Jersey continental slope: Implications for slope failure and cold seeps. *Science*, **289**(5477), 288-291. (2000).

10. Bethke, C.M. and Corbet, T.F. Linear and nonlinear solutions for one-dimensional compaction flow in sedimentary basins. *Water Resources Research*, **24**(3), pp.461-467. (1988).

11. Neuzil, C. E. Groundwater flow in low-permeability environments. *Water Resources Research*, **22**(8), 1163-1195. (1986).

12. Goodbred Jr, S. L., & Kuehl, S. A. The significance of large sediment supply, active tectonism, and eustasy on margin sequence development: Late Quaternary stratigraphy and evolution of the Ganges–Brahmaputra delta. *Sedimentary Geology*, **133**(3-4), 227-248. (2000).

13. Bethke, C.M., Reed, J.D. and Oltz, D.F. Long-range petroleum migration in the Illinois Basin. *AAPG Bulletin*, **75**(5), pp.925-945. (1991).
