# Supplementary material for: Author Correction: Buried deep freshwater reserves beneath salinity-stressed coastal Bangladesh
Source: Nat Commun. 2026 Feb 4;17:1321. doi: 10.1038/s41467-026-69228-z (PMC12873189; doi:10.1038/s41467-026-69228-z)
Supplement: Supplementary file 1 — Original Fig. 2 [file 41467_2026_69228_MOESM1_ESM.pdf]

Original, uncorrected Fig. 2

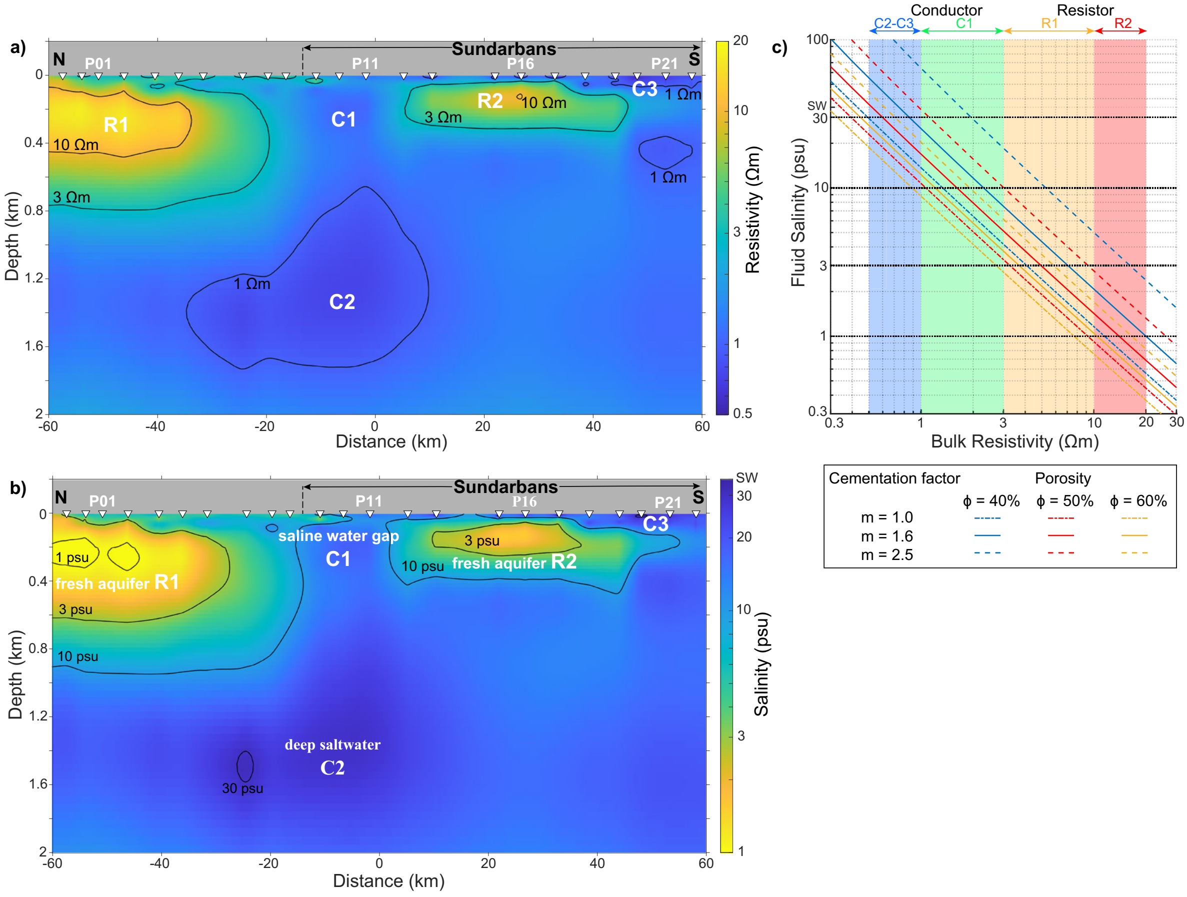

Fig. 2 | Preferred resistivity and salinity models. a 2D electrical resistivity model derived from inversion of magnetotelluric data. Yellow/warm colors represent resistive features (up to 20 Ωm), associated with freshwater zones (R1, R2). Blue/cold colors indicate conductive features (0.5–2 Ωm), associated with saline zones (C1, C2, C3). The 1 and 10 Ωm contours delineate the boundaries of conductive and resistive zones, respectively, with the 3 Ωm contour marking the transition between them. **b** The corresponding 2D salinity model estimated using Archie's law and the Practical Salinity Scale 1978 (PSS78; unit: practical salinity unit) (see Methods). Yellow/warm colors represent fresh, low-salinity regions. Blue/cold colors indicate saline, high-salinity regions. The 3 psu contour defines the freshwater boundary, while the 10 psu contour marks the transition between low- and high-salinity brackish water. The northern freshwater aquifer (R1) and the southern aquifer within the Sundarbans (R2) are separated by a saline water gap (C1). Color scales in (**a**) and (**b**) are logarithmic ($\log_{10}$), but values are labeled in linear units. **c** Pore fluid salinity varies with bulk resistivity, porosity and cementation factors. Salinity is calculated as a function of bulk resistivity (ρ_b), porosity (ϕ), and cementation factor (m) by using Archie's law[68] and PSS78[70] with fixed pressure and temperature conditions: P = 0 dbars and T = 30 °C. The color bands denote resistive and conductive zones in Fig. 2a. Porosity and cementation factor values illustrate different lithological conditions of sediments.
